# Survival among rectal cancer patients in Belgium receiving different preoperative and postoperative treatment: a population-based comparison

**DOI:** 10.1186/2049-3258-73-S1-P7

**Published:** 2015-09-17

**Authors:** Geert Silversmit, Tamara Vandendael, Ines Joye, Annelies Debucquoy, Freddy Penninckx, Evelien Vaes, Karin Haustermans

**Affiliations:** 1 Belgian Cancer Registry, Brussels, Belgium; 2 University Hospitals Leuven, Leuven, Belgium; 3 University of Leuven, Leuven, Belgium

## Introduction

About 1500 men and 1000 women are yearly diagnosed with rectal cancer in Belgium. Total mesorectal excision surgery is regarded as the cornerstone for rectal cancer treatment. In addition, (chemo)radiotherapy is often administered prior to surgery and adjuvant chemotherapy afterwards.

## Study goals

Postoperative observed survival was compared on a population-based level among clinical stage I-III rectal cancer patients who were diagnosed in 2006-2011 and received either preoperative radiotherapy, chemoradiotherapy or no preoperative therapy. Adjuvant chemotherapy and type of radical resection were also considered as clinical outcomes.

## Methods

Details on surgery and pre/postoperative therapy were obtained by coupling the Belgian Cancer Registry records with the Belgian medical claims (IMA) database. Cox regression models were applied to adjust for age, clinical stage, gender and WHO score. As adjuvant chemotherapy is administered after surgery, patients having received adjuvant chemotherapy experience the so called ‘immortal time bias’. Therefore, adjuvant chemotherapy was modelled as a time-dependent covariate to take into account the time delay between surgery and the start of the adjuvant chemotherapy.

## Results

A cohort of 5173 rectal cancer patients was followed until the 1^st^ of October 2014. Higher postoperative observed survival was found for the patient group (1) receiving preoperative chemoradiotherapy compared to no preoperative therapy or preoperative radiotherapy alone, although the hazard ratio depends on age, (2) that did not receive adjuvant chemotherapy versus those who did and (3) that underwent a sphincter saving radical resection versus those who underwent an abdominoperineal excision, although the hazard ratio depends strongly on age. Similar differences between the same patient groups were also obtained for the observed survival conditional on surviving the first year since surgery.

## Conclusions

In this population-based study, preoperative chemoradiotherapy, no adjuvant chemotherapy and sphincter saving operations were associated with a superior survival for clinical stage I-III rectal cancer patients.

